# A nomogram for predicting choledochal cyst with perforation

**DOI:** 10.1007/s00383-024-05710-3

**Published:** 2024-05-10

**Authors:** Guangwei Zhang, Haoming Wang, Jianyang Hu, Chenyu Yang, Bingqian Tan, Jiqiang Hu, Mingman Zhang

**Affiliations:** Department of Hepatobiliary Surgery, Children’s Hospital of Chongqing Medical University, Chongqing Key Laboratory of Pediatrics, National Clinical Research Center for Child Health and Disorders, Ministry of Education Key Laboratory of Child Development and Disorders, Chongqing, China

**Keywords:** Choledochal cyst, Perforation, Nomogram

## Abstract

**Background:**

Choledochal cyst with perforation (CC with perforation) rarely occurs, early diagnosis and timely treatment plan are crucial for the treatment of CC with perforation. This study aims to forecast the occurrence of CC with perforation.

**Methods:**

All 1111 patients were conducted, who underwent surgery for choledochal cyst at our hospital from January 2011 to October 2022. We conducted univariate and multivariate logistic regression analysis to screen for independent predictive factors for predicting CC with perforation, upon which established a nomogram. The predictive performance of the nomogram was evaluated using receiver operating characteristic (ROC) curves, calibration plots, and decision curve analysis (DCA) curves.

**Results:**

The age of children with choledochal cyst perforation is mainly concentrated between 1 and 3 years old. Logistic regression analysis indicates that age, alanine aminotransferase, glutamyl transpeptidase, C-reactive protein, vomiting, jaundice, abdominal distension, and diarrhea are associated with predicting the occurrence of choledochal cyst perforation. ROC curves, calibration plots, and DCA curve analysis curves demonstrate that the nomogram has great discriminative ability and calibration, as well as significant clinical utility.

**Conclusion:**

The age of CC with perforation is mainly concentrated between 1 and 3 years old. A nomogram for predicting the perforation of choledochal cyst was established.

## Introduction

Choledochal cyst (CC) is a common type of biliary malformation in infants and young children, characterized by segmental dilation of the intrahepatic and extrahepatic bile ducts in a cylindrical, cystic, or fusiform shape [1]. It has a higher incidence rate in Asian people, and the incidence rate of CC in Asian people is 1:1000 [2].

CC with perforation is a rare condition [3], and the mechanism of choledochal cyst perforation is not yet clear. The current understanding suggests that it is caused by the reflux of pancreatic enzymes, increased pressure in the bile duct due to protein blockage, and viral infection [4].

CC with perforation, as an acute abdomen, requires immediate surgical intervention. Delayed diagnosis or improper management can lead to complications such as bile peritonitis, bacterial contamination, sepsis, cholangitis and pancreatitis. However, there is no unified treatment guideline for CC with perforation. And, the mainstream approach often involves a two-stage surgical treatment, after the initial surgery, the choledochal cyst is typically removed within 1–3 months [5].

## Materials and methods

### Study design and patients

Following institutional review board approval. A retrospective analysis was conducted on the electronic medical records of patients who underwent choledochal cyst excision with hepaticojejunostomy at the Department of Hepatobiliary Surgery, Children’s Hospital Affiliated to Chongqing Medical University, from January 2011 to October 2022. The study included 1111 patients (63 with perforation, 1048 without perforation), and all patients with perforation were confirmed during intraoperative examination. All surgeries were performed by the same experienced surgical team. Variables including demographic data, symptoms, and serum biochemical markers were collected and analyzed.

### Inclusion criteria

Inclusion criteria were as follows: (1) Patients diagnosed with choledochal cyst (CC) based on intraoperative findings and postoperative pathological results; (2) Patients who received surgical treatment at the Children’s Hospital Affiliated to Chongqing Medical University as their primary hospital; (3) Patients with no history of previous abdominal surgery; (4) Confirmation of CC with perforation or cystic necrotic changes.

### Variable definition

Predictive variables are categorized into demographic variables and clinical data variables. Demographic variables include age and gender at the time of presentation. Clinical data variables encompass signs and biochemical data at the time of presentation. The signs include strong yellow urine, acholic stools, abdominal pain, vomiting, high TBIL level (defined as total bilirubin ≥17.1 µ/L for diagnosis of subclinical jaundice), fever, abdominal distension, diarrhea, and the presence of gallstones. Biochemical data include total bilirubin (TBIL), direct bilirubin (DBIL), indirect bilirubin (IBIL), aspartate aminotransferase (AST), alanine aminotransferase (ALT), lactate dehydrogenase (LDH), alkaline phosphatase (ALP), gamma-glutamyl transferase (GGT), C-reactive protein (CRP, when the C-reactive protein in our research center is less than 8 mg/L, the test reports all show 8 mg/L.), and AST/ALT ratio (AST/ALT).

### Statistical analysis

Data with homogeneity of variance were subjected to statistical analysis using paired t-test and expressed as mean ± standard deviation. Data with inhomogeneity of variance were analyzed using Student’s two-tailed t-test and expressed as median ± interquartile range (IQR).

Categorical variables are described using ‘frequency’ and the differences between groups of categorical variables are analyzed using chi-square test. Single-factor and multi-factor analysis are used to screen prognostic factors, and variables with statistically significant inter-group differences in multi-factor logistic regression analysis (*p* < 0.05) are defined as independent prognostic factors. Nomograms are constructed using independent prognostic factors. Receiver operating characteristic (ROC) curves are plotted using training and validation set data, and the area under the curve (AUC) is calculated to assess the model’s discriminative ability; calibration curves are plotted to assess the model’s accuracy, decision curve analysis (DCA) is used to assess the clinical applicability of the model [6].

This study is a retrospective study. Due to variations in detection instruments among different treatment centers, there is bias in the baseline data. Therefore, this predictive model is not suitable for external validation. As a result, the 1111 patients collected will be randomly divided into a test group and a validation group in a 7:3 ratio randomly. The statistical software used in this study includes SPSS 27.0, GraphPad Prism 8.1, and R software, with R programming packages such as car, rms, proc and rmda.

## Result

### Analysis of clinical data and biochemical indicators

**Fig. 1:**
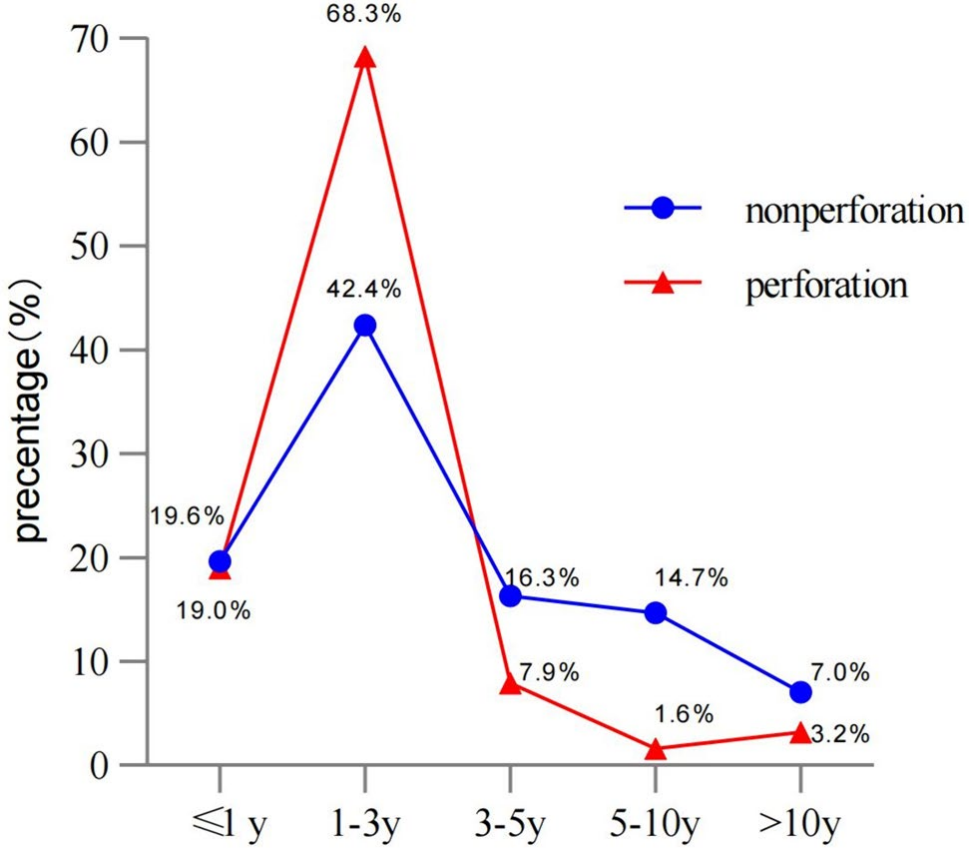
Age distribution chart for the perforation and non-perforation groups

This study included a total of 1111 patients with choledochal cysts (267 males and 844 females), among whom 63 (13 males and 50 females) had perforations (perforation group) and 1048 (264 males and 794 females) did not have perforations (non-perforation group). Statistical analysis of the clinical data of the two groups revealed that the average age of the perforation group was lower than that of the non-perforation group [21 (13.3–36.0) vs. 24 (15–48), *p* < 0.01], and their age distribution is shown in Fig. 1. Additionally, the chi-square test showed that the perforation group had a higher frequency of vomiting [Perforation (85.7%) vs. non-perforation (55.0%)], jaundice [Perforation (42.9%) vs. non-perforation (32.7%)], fever [Perforation (55.6%) vs. non-perforation (18.2%)], abdominal distension [Perforation (57.1%) vs. non-perforation (20.2%)] and diarrhea [Perforation (19.0%) vs. non-perforation (2.1%)] compared to the non-perforation group. (Data are shown in Table 1).

**Table 1:** Demographic and symptom of non-perforation group and perforation group

	Perforation (63)	Non-perforation (1048)	*p* value
Sex (male: female)	13:50	264:794	*x*^2^ = 0.44 *p* = 0.516
Age (month)	24.0 (15.0–48.0)	21.0 (13.3–36.0)	*p* < **0.001**
Abdominal pain (person)	42	686	*x*^2^ = 0.038 *p* = 0.845
Vomiting (person)	54	577	*x*^2^ = 22.762 *p* < **0.001**
High TBIL level (person)	27	343	*x*^2^ = 67.366 *p* < **0.001**
Acholic stool (person)	14	248	*x*^2^ = 0.131 *p* = 0.937
Fever (person)	35	190	*x*^2^ = 51.54 *p* < **0.001**
Abdominal distension (person)	36	212	*x*^2^ = 46.702 *p* < **0.001**
Diarrhea (person)	12	22	*x*^2^ = 57.541 *p* < **0.001**
Bile duct stones (person)	8	254	*x*^2^ = 4.39 *p* = **0.036**

Bold values denote the *p* values < 0.05

In the statistical analysis of the biochemical data, the levels of TBIL [45.7 ± 41.8 vs. 25.7 ± 45.07), *p* < 0.001], IBIL [25.4 (12.5–39.0) vs. 6.6 (3.2–16.0), *p* < 0.001], AST [95.6 (77.6–166.4) vs. 48.0 (34.2–70.8), *p* < 0.001], ALT [93.4 (72.1–175.7) vs. 44.2 (26.4–72.6), *p* < 0.001], LDH [363.3 (212.1–331.8) vs. 238.9 (203.0–287.0), *p* = 0.088], GGT [364.5.7 (9.3–1116.0) vs. 190.2 (9.3–3126.0), *p* = 0.023], ALP [387.8 (300.9–514.0) vs. 254.2 (187.9–414.9), *p* = 0.043] and CRP [23.6 (8.0–90.0) vs.11.0 (8.0–180.0), *p* < 0.001] were higher in the perforation group compared to the non-perforation group. (Data are shown in Table 2).

**Table 2:** Biochemical index of non-perforation group and perforation group

Biochemical index	Perforation	Non-perforation	*p* value
TBIL (normal range: 1.8–21 µ/L)	45.7 ± 41.8	25.7 ± 45.07	**0.004**
DBIL (normal range: 0–6.7 µ/L)	3.4 (1–13.9)	2.2 (1–7.5)	0.741
IBIL (normal range: 0–19.5 µ/L)	25.4 (12.5–39.0)	6.6 (3.2–16.0)	**0.006**
AST (normal range: 0–45 µ/L)	95.6 (77.6–166.4)	48.0 (34.2–70.8)	**<0.001**
ALT (normal range: 0–40 µ/L)	93.4 (72.1–175.7)	44.2 (26.4–72.6)	**<0.001**
AST/ALT (normal range: 0.23–2.47)	1.11 (0.90–1.36)	1.18 (0.78–1.17)	0.217
LDH (normal range: 110–330 µ/L)	363.3 (212.1–331.8)	238.9 (203.0–287.0)	**0.023**
ALP (normal range: 95–405 µ/L)	387.8 (300.9–514.0)	254.2 (187.9–414.9)	**0.043**
GGT (normal range: 5–38 µ/L)	477.1 ± 213.3	183.9 ± 203.7	**<0.001**
CRP (normal range: 0–8 mg/L)	23.6 (8.0–90.0)	11.0 (8.0–180.0)	**0.001**

Bold values denote the *p* values < 0.05

Biochemical data include total bilirubin (TBIL), direct bilirubin (DBIL), indirect bilirubin (IBIL), aspartate aminotransferase (AST), alanine aminotransferase (ALT), lactate dehydrogenase (LDH), alkaline phosphatase (ALP), gamma-glutamyl transferase (GGT), C-reactive protein (CRP, when the C-reactive protein in our research center is <8 mg/L, the test reports all show 8 mg/L.), and AST/ALT ratio (AST/ALT)

### ROC curve

**Fig. 2:**
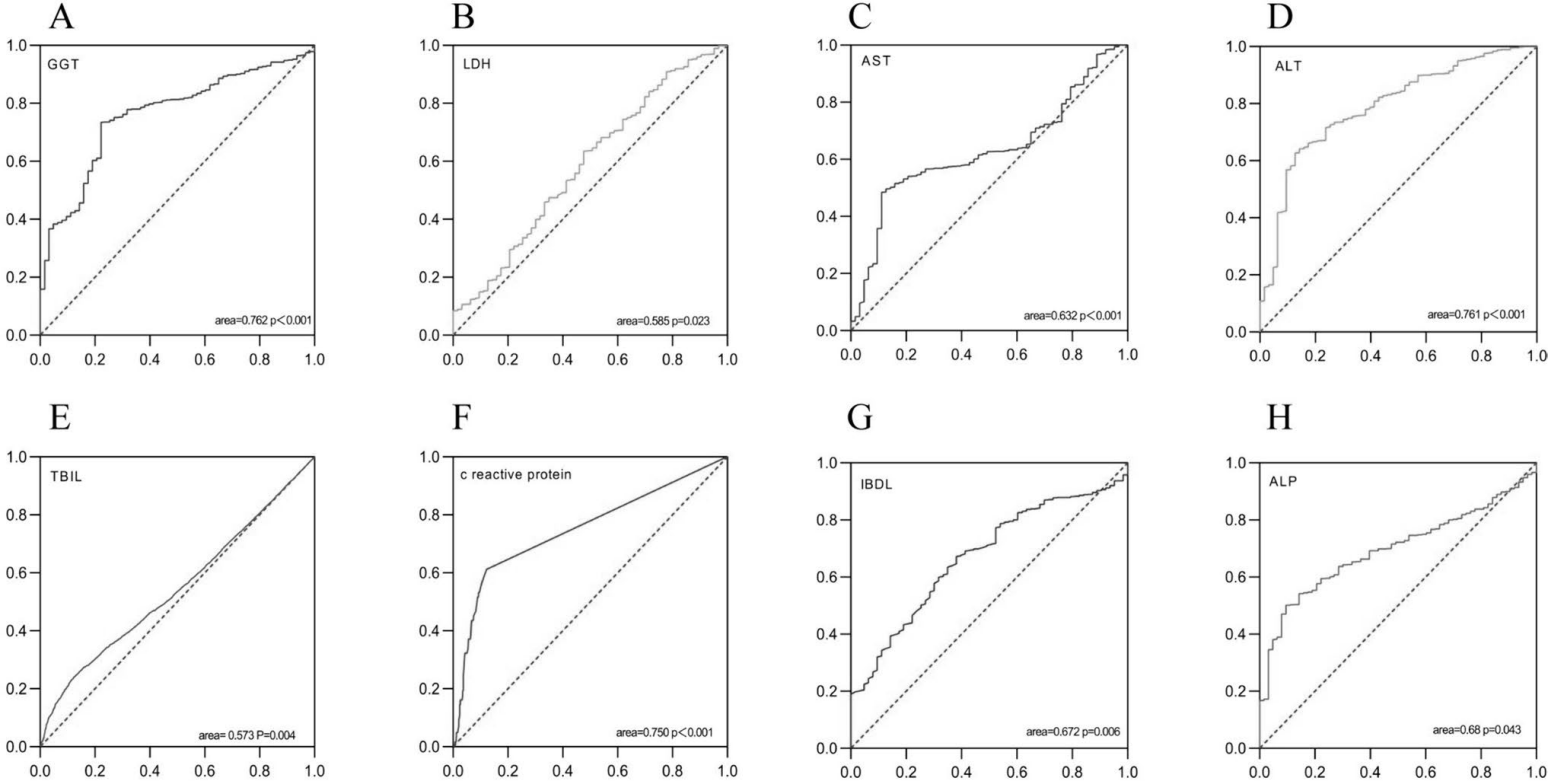
Perform single factor ROC curve analysis on 8 meaningful indicators [GGT, LDH, AST, ALT, TBIL, CRP, IBDL, ALP] from (**A–H**). The AUC are 0.762, 0.585, 0.632, 0.761, 0.573, 0.750, 0.672 and 0.681

We included serum indicators with intergroup differences in the ROC analysis. The statistically significant indicators include: GGT, LDH, AST, ALT, TBIL, CRP, IBDL, ALP, and their AUC are 0.762, 0.585, 0.632, 0.761, 0.573, 0.750, 0.672 and 0.681. (Shown in Fig. 2).

### Collinearity analysis and logistic regression analysis

We incorporated all predictive factors from the text set into the univariate logistic regression analysis. Among them, age, TBIL, DBIL, IBIL, AST, ALT, LDH, ALP, GGT, CRP, vomiting, jaundice, fever, abdominal distension, diarrhea had statistical significance (*p* < 0.05). Then, we incorporated all factors with statistical significance in univariate logistic regression analysis into the multivariate logistic regression analysis, it suggested that age, ALT, GGT, CRP, vomiting, abdominal distension, diarrhea, and jaundice are independent predictive factors influencing CC with perforation (*p* < 0.05).

To investigate multicollinearity among all continuous independent predictive factors, we included all independent predictive factors in a collinearity analysis. All continuous independent predictive factors’ VIF < 5. (All data are shown in Table 3).

**Table 3:** Logistic regression analysis and collinearity analysis in the text dataset

Index	Univariate analysis		multivariate analysis		Collinearity analysis
	Exp (B) (95% CI)	*p*	Exp (B) (95% CI)	*p*	VIF
Sex	0.847 (0.399–1.797)	0.665			
Age	0.983 (0.970–0.996)	**0.009**	0.968 (0.949–0.987)	**0.001**	1.05
TBIL	1.010 (1.004–1.015)	**<0.001**	1.097 (0.910–1.322)	0.332	
DBIL	1.010 (1.001–1.020)	**0.037**	0.888 (0.736–1.072)	0.216	
IBIL	1.015 (1.006–1.023)	**<0.001**	0.908 (0.753–1.096)	0.317	
AST	1.010 (1.006–1.012)	**<0.001**	1.002 (0.996–1.008)	0.468	
ALT	1.014 (1.010–1.019)	**<0.001**	1.013 (1.005–1.020)	**0.001**	2.81
LDH	1.003 (1.001–1.005)	**0.003**	0.999 (0.994–1.004)	0.689	
ALP	1.001 (1.000–1.002)	**0.030**	1.000 (0.999–1.002)	0.602	
GGT	1.004 (1.002–1.005)	**<0.001**	1.003 (1.002–1.004)	**<0.001**	1.61
CRP	1.026 (1.013–1.039)	**<0.001**	1.033 (1.014–1.053)	**<0.001**	1.01
Abdominal pain	0.911 (0.484–1.716)	0.774			
Vominting	4.218 (1.856–9.586)	**<0.001**	8.722 (2.237–30.314)	**0.002**	
Jaundice	7.368 (3.791–14.594)	**<0.001**	8.722 (2.813–27.040)	**<0.001**	
Acholic stool	1.034 (0.512–2.088)	0.926			
Strong yellow urine	0.935 (0.487–1.797)	0.841			
Fever	5.695 (3.049–10.638)	**<0.001**	1.406 (0.514–3.849)	0.507	
Abdominal distension	4.962 (2.662–9.248)	**<0.001**	4.665 (1.825–11.925)	**<0.001**	
Diarrhea	13.125 (5.544–31.071)	**<0.001**	42.024 (9.943–177.6122)	**<0.001**	

Bold values denote the *p* values < 0.05

### Nomogram construction

**Fig. 3:**
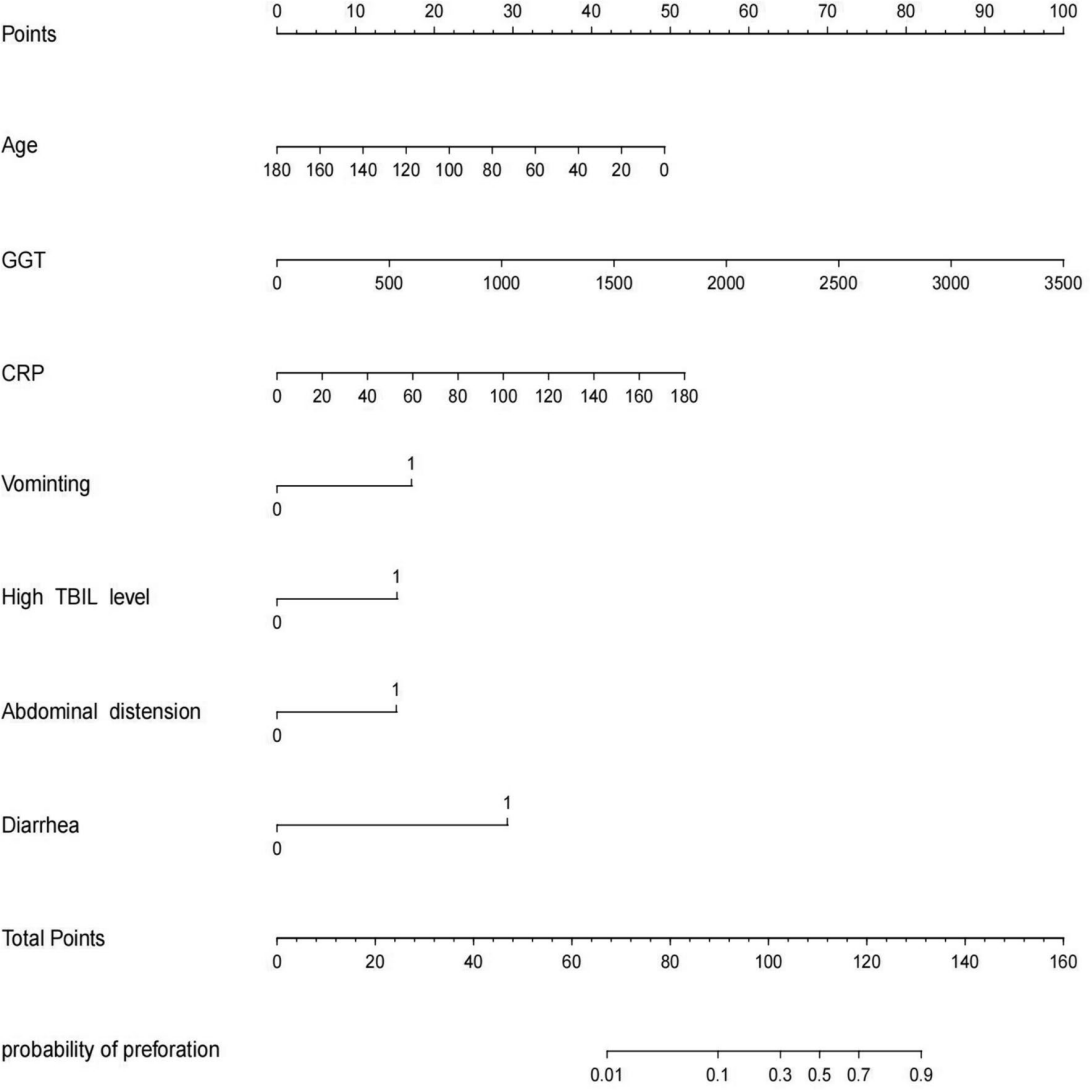
Nomogram predict the likelihood of perforation in patients with choledochal cysts. To determine the points received for each level, *draw a line upwards* on each variable axis. Add up the scores for each category to get the total score and mark it on the “Total Points” axis. *Draw a line down* from each point to the probability axis to determine the likelihood of perforation in patients with choledochal cysts. (0 indicates the absence of the symptom, while 1 indicates the presence of the symptom)

**Fig. 4:**
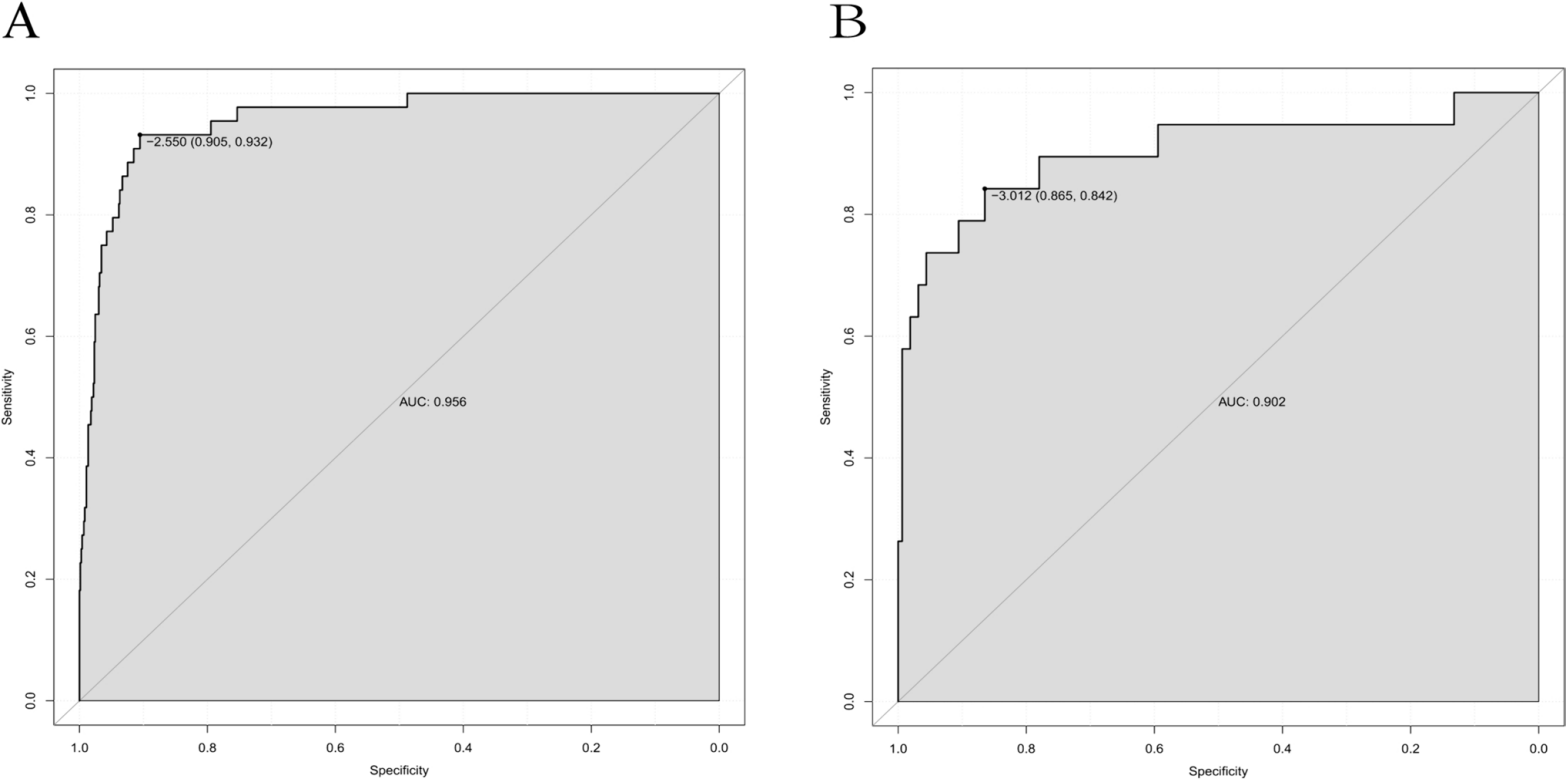
ROC curves of the nomogram. **A** ROC curves in the text set. **B** ROC curves in the validation set

**Fig. 5:**
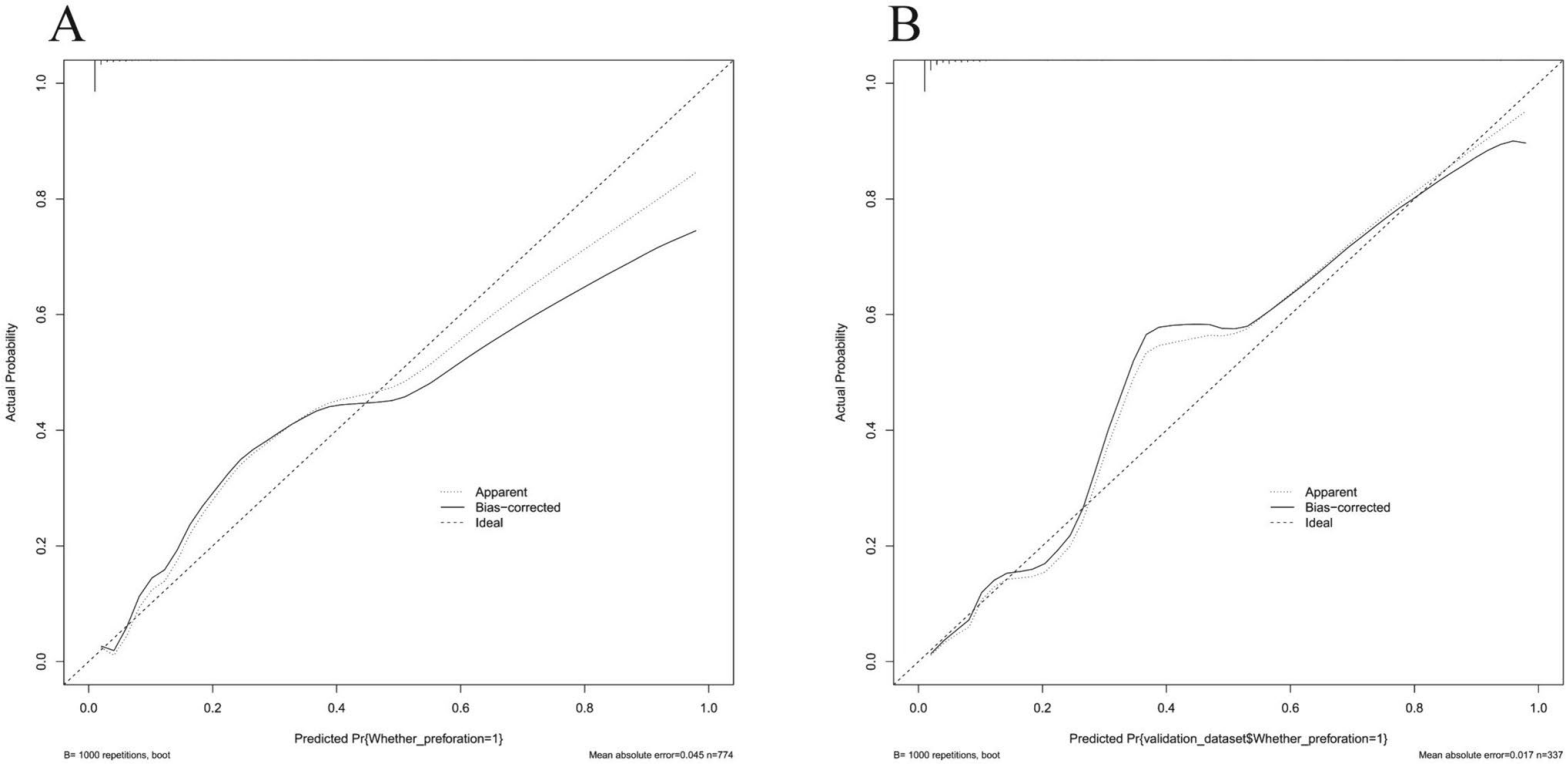
Calibration curves of the nomogram **A** Calibration curves in the text set. **B** Calibration curves in the validation set

**Fig. 6:**
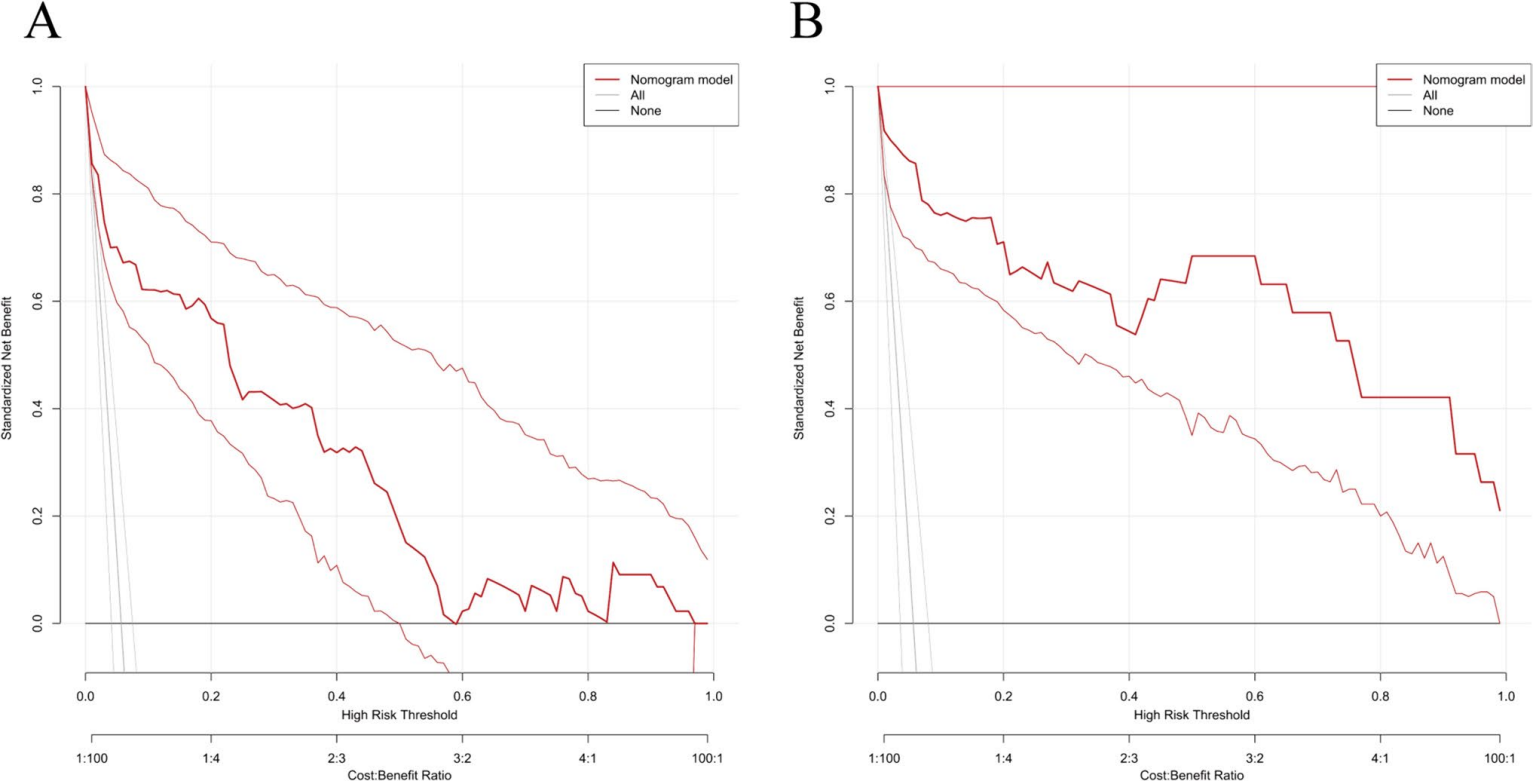
Decision curves analysis (DCA) plots of the nomogram. **A** DCA plots in the text set. **B** DCA plots in the validation set

The independent factors affecting the above-mentioned predictions are used to construct a nomogram. As shown in Fig. 3, CC patients can obtain personalized scores from our nomogram and correspond to their predicted probabilities of perforation. In the test set, the AUC of the ROC curve of the nomogram is 0.956 (Fig. 4A). In the validation set, the AUC is 0.902 (Fig. 4B), indicating that the model has a great performance in predicting CC with perforation. Calibration curves for the test set and validation set shown that the calibration curve is relatively evenly distributed around the ideal line (calibration curve/ideal curve), indicating great consistency between the nomogram’s predictions and the actual results (Fig. 5A and B). Decision plots (DCA) in the test and validation sets demonstrate that our predictive model has great clinical applicability (Fig. 6A and B).

## Discussion

CC with perforation with is a relatively rare complication, and the consequences of misdiagnosis and delayed treatment can be fatal [7]. Due to the lack of typical clinical manifestations, preoperative diagnosis of CC with perforation is challenging [7]. In this study, we aim to summarize the personal data, clinical signs, and biochemical data of these patients, and through logistic regression analysis, in order to facilitate early detection of perforation.

The exact pathogenesis of choledochal cyst perforation remains controversial [8]. Based on literature review and clinical experience, we thought that the mechanisms of CC with perforation may be the following reason. First, abnormal pancreaticobiliary junction leads to retrograde flow of pancreatic fluid into the common bile duct through a common channel. Pancreatic proteases are activated by intestinal enzymes, causing destruction of the cyst wall and resulting in perforation [9]. Second, cystic dilatation of the distal common bile duct is prone to protein plug formation, leading to acute obstruction and a rapid increase in biliary pressure, resulting in perforation [10]. Third, it is associated with inadequate development of biliary blood supply and elastic fibers [11, 12]. Fourth, in cases of common bile duct cysts with concurrent infection, it can cause edema, mucosal ulceration, and necrosis of the bile duct wall, leading to perforation [13]. In our previous clinical surgical experience, the second and fourth points mentioned are the most common causes.

In this study, we developed a nomogram to predict the occurrence of perforated choledochal cysts, in which age, ALT, GGT, CRP, vomiting, abdominal distension, diarrhea, and jaundice are independent predictive factors influencing CC with perforation. The exacerbation of biliary obstruction in the occurrence of choledochal cyst perforation suggests that GGT, as an indicator reflecting the degree of biliary obstruction [14], can be expected to be an independent predictive factor for choledochal cyst perforation. We also thought that the occurrence of perforation leads to the overflow of bile from the perforated site, which triggers a strong inflammatory response, resulting in acute symptoms such as abdominal pain, fever [15], and elevated CRP levels. Additionally, bile stimulation of the intestinal wall may lead to intestinal distension and abdominal fluid accumulation, increasing the probability of abdominal distension in perforated patients [16]. Similarly, for the same reasons, the probability of diarrhea occurrence in patients may also increase.

In related literature, we found that the diagnostic feature of CC with perforation is the interruption of bile duct continuity on ultrasound, with a specificity of 100%. However, the sensitivity of ultrasound detection is relatively low (18.6%) [17]. Local loss of gallbladder tension, thickening of the gallbladder wall, abnormal shape of the gallbladder wall, and ascites can also be used as imaging features of CC with perforation [17]. There is also evidence indicating that in emergency situations, CT can be helpful in predicting choledochal cyst perforation, particularly for abnormalities in the retroperitoneal space [12, 18, 19]. Similarly, MRCP is a valuable diagnostic tool that can non-invasively display anomalies in the biliary and pancreatic ducts. Although MRCP has superior soft tissue resolution and multiplanar capabilities [3, 20, 21], some studies suggest that it may be challenging to accurately identify discontinuities in the biliary duct wall. However, it can specifically assess the severity of biliary peritonitis, including pseudo-cysts around the liver, providing significant value in evaluating the condition of perforated patients. However, due to the acute onset of perforation in patients, emergency surgical treatment is often required, making it difficult for many perforated patients to undergo MRCP examination promptly, leading to a loss of imaging data. Furthermore, related studies have found that non-cystic bile ducts are a risk factor for bile duct perforation [22]. Ando et al [13], also reported that in cases of bile duct perforation, the extrahepatic bile ducts were spindle-shaped.

For surgical treatment of choledochal cyst perforation, there are currently two main approaches, including single-stage and two-stage surgical treatments. In our previous clinical experience, 50 perforation patients underwent two-stage surgery, while 13 patients underwent single-stage surgery. We also found that perforation patients can even undergo choledochal cyst excision under laparoscopy.

There is a difference in the choice between two-stage and single-stage surgeries as they each have their own advantages and disadvantages. The advantages of radical surgery include the need for only one surgery and potential cost-effectiveness [23]. In addition, patients undergoing two-stage surgery, after undergoing external biliary drainage, the adhesions in the abdominal cavity during the second-stage surgery are usually more tightly bound than those in the first-stage surgery [24], which increases the anatomical difficulty of the surgery and the risk of iatrogenic injury, as well as the probability of postoperative complications.

The limitations of this study include its retrospective nature and the fact that many perforation cases were acute in nature, with some medical records being of a distant nature. This resulted in a lack of complete initial imaging results to refine our model for predicting perforated choledochal cysts, potentially leading to selection bias. Furthermore, due to variations in diagnostic equipment across different medical centers, our model underwent only internal validation rather than external or prospective validation, which may affect its applicability. Our future goal is to evaluate the performance of the nomogram through prospective external validation and provide treatment guidance for patients with perforated choledochal cysts based on the nomogram’s results.

## Conclusion

By collecting medical records of patients with choledochal cyst perforation, we have proposed our ideas for the diagnosis and treatment of these patients and established a predictive model. However, the model still needs to be improved. At the same time, we also believe that single-stage surgery is feasible for patients with perforation, but it should not be blindly chosen. Whether to perform single-stage or two-stage surgery depends on the patient’s physical condition and tolerance.

## Data Availability

The datasets generated during and analyzed during the current study are available from the corresponding author on reasonable request.
